# Depth of implant mucosal tunnel influences its microbiota: a prospective observational cross-sectional study

**DOI:** 10.1186/s12903-026-09312-4

**Published:** 2026-07-17

**Authors:** Igor Ashurko, Svetlana Bokareva, Oxana Svitich, Svetlana Tarasenko, Dmitry Kompaniets, Asmik Avagyan, Nune Vartanova, Mamedova Rasana, Alexey Unkovskiy

**Affiliations:** 1 Department of Oral Surgery, E.V. Borovsky Institute of Dentistry, Sechenov First Moscow State Medical University, Moscow, 119048 Russia; 2 La Metamorphose, Moscow, 121151 Russia; 3 Department of Microbiology, Virology, and Immunology, Sechenov First Moscow State Medical University, Moscow, 125009 Russia; 4 Mechnikov Research Institute of Vaccines and Sera, Moscow, 105064 Russia; 5 Department of Prosthodontics, Geriatric Dentistry and Craniomandibular Disorders, Charité - Universitätsmedizin Berlin, Aßmannshauser Str. 4-6, Berlin, 14197 Germany

**Keywords:** Peri-implant tissue, Implant mucosal tunnel, Supracrestal soft tissue, Microbiota, Immune response, *TNF-α*

## Abstract

**Purpose:**

to assess whether implant mucosal tunnel depth is associated with differences in cultivable microbial diversity in clinically healthy peri-implant tissues.

**Methods:**

fifty-two patients with single molar implants were included after a standardized 3-month healing period and stratified into three groups according to implant mucosal tunnel depth: <3 mm, 3–5 mm, and > 5 mm. Microbial samples from the inner lining of the implant mucosal tunnel were cultured under aerobic and anaerobic conditions and identified using MALDI-TOF mass spectrometry. As a secondary exploratory outcome, TNF-α expression in peri-implant mucosa was quantified using real-time PCR.

**Results:**

a total of 262 cultivable isolates representing 46 species were identified, with facultative anaerobic species predominating (81.68%). The highest Shannon diversity index was observed in the 3–5 mm group, whereas tunnels > 5 mm demonstrated reduced cultivable diversity, with clinically significant growth predominantly represented by streptococci. TNF-α expression showed a progressive increase with increasing implant mucosal tunnel depth, reaching approximately 38-fold higher levels in the > 5 mm group compared with < 3 mm (*p* < 0.017).

**Conclusions:**

within the limitations of this exploratory cross-sectional study, implant mucosal tunnel depth was associated with differences in cultivable microbial composition and local TNF-α expression in clinically stable peri-implant tissues. Deeper mucosal tunnels (> 5 mm) demonstrated reduced cultivable species diversity without a consistent predominance of cultivable periodontal pathogens under the applied culture conditions. The observed increase in TNF-α expression should be interpreted as a biologic association rather than evidence of active inflammation or disease. Due to the culture-based methodology and the absence of longitudinal clinical data, no conclusions regarding disease risk or progression can be drawn.

**Trial registration:**

ClinicalTrials.gov, NCT05870774. Registered on 20,230,425.

## Background

The condition of peri-implant soft tissues constitutes a critical factor influencing the efficacy of implant-supported prostheses and long-term implant survival rate [1–7]. Adequate soft tissue volume enables masking of metallic implant suprastructure components and provides the spatial foundation for anatomically contoured prosthetic crowns. Contemporary evidence further indicates that implant platform positioning relative to the soft tissue margin correlates with biological complications [8–13]. It has been suggested that excessive deepening of the implant mucosal tunnel may create an unfavorable environment for biofilm removal around implant restorations, particularly when combined with prosthetic contour characteristics [14]. Biofilm is known to penetrate deeply into the mucosal tunnel along prosthetic components, with microbial colonization commencing as early as 30 min post-implant placement [15]. Multiple authors emphasize that accessibility for biofilm debridement around implant crowns is critical for preventing and managing inflammatory peri-implant diseases and bone stability [16–18].

It has been shown that the efficacy of self-performed oral hygiene is reduced with increasing probing depth measured from the mucosal margin [17]. Clinical studies indicate that a high percentage of implants diagnosed with peri-implantitis correlate with inadequate biofilm control or limited accessibility for oral hygiene measures [18]. Evidence suggests that increased peri-implant mucosal tunnel depth may be associated with differences in subgingival microbial composition and has been linked to a higher prevalence of peri-implant inflammatory conditions [19, 20]. This is particularly relevant for patients with a history of periodontitis, as excessive vertical soft tissue thickness in this cohort adversely affects peri-implant tissue health according to Zhang et al. (2020), who demonstrated that each 1-mm increase in soft tissue thickness corresponded to a 1.5-fold higher risk of peri-implantitis [21].

Despite extensive research on peri-implant microbiota, comparative analysis of bacterial profiles around implants with varying vertical dimension of the implant mucosal tunnel remains unexplored. Current literature provides no consensus regarding the maximum vertical dimension of the implant mucosal tunnel that ensures long-term tissue health [22–27]. Therefore, the present study aims to clarify if the depth of implant mucosal tunnel may influence its microbiota.

In addition, biological markers may provide deeper insight into peri-implant tissue conditions. TNF-α was selected as the target pro-inflammatory cytokine due to its well-documented role in early inflammatory signaling at the peri-implant interface and its involvement in host immune responses and bone metabolism [28–30]. 

## Methods

### Patients’ recruitment and study design

This study was designed as a prospective observational study with cross-sectional analysis and was conducted from 2021 to 2024 at the Department of Surgical Dentistry and Maxillofacial Surgery, I.M. Sechenov First Moscow State Medical University (Sechenov University), Moscow, Russian Federation. No intervention, randomization, or investigator-driven allocation was performed; patients were stratified into study groups after the healing period according to the measured depth of the implant mucosal tunnel.

The study protocol was approved by the Local Ethics Committee of I.M. Sechenov First Moscow State Medical University (approval No. 01–21 dated 22.01.2021). All participants provided written informed consent prior to enrollment. All procedures involving human participants were carried out in accordance with the ethical standards of the institutional research committee and with the 1964 Declaration of Helsinki and its later amendments. The study protocol was prospectively registered at ClinicalTrials.gov (Identifier: NCT05870774). The manuscript was prepared with consideration of the STROBE recommendations for reporting observational studies.

The primary objective of the study was to assess whether implant mucosal tunnel depth is associated with differences in the cultivable peri-implant microbiota in clinically stable peri-implant tissues. The secondary objective was to evaluate local TNF-α gene expression in relation to implant mucosal tunnel depth as an exploratory marker of peri-implant biological activity.

The working hypothesis assumed that implant mucosal tunnel depth may influence the composition of cultivable peri-implant microbiota. An additional exploratory hypothesis was that implants with mucosal tunnel depth greater than 5 mm may demonstrate distinct microbiological and immunological characteristics compared with shallower soft tissue tunnels.

The inclusion criteria were as follows: patients older than 20 years who had signed informed consent for participation, with an ASA physical status of I or II according to the American Society of Anesthesiologists classification, presenting with a single missing first or second molar in either jaw and not requiring bone augmentation procedures. Additional requirements included horizontal soft tissue thickness greater than 2 mm and keratinized mucosa width greater than 2 mm at the edentulous site, full-mouth bleeding score (FMBS) and full-mouth plaque score (FMPS) both below 20%, absence of bleeding on probing, probing depth of the residual dentition not exceeding 3 mm, no clinical or radiographic signs of active periodontal inflammation, satisfactory oral hygiene, and high patient motivation to maintain oral hygiene and adhere to study protocols.

The exclusion criteria comprised uncompensated systemic conditions, heavy smoking (more than 10 cigarettes per day), previous bone grafting at the intended implant site, probing depth exceeding 4 mm at residual dentition, acute or decompensated comorbidities, a history of radiotherapy or chemotherapy within the previous five years, pregnancy or lactation, and the current use of medications known to impair soft tissue healing, including non-steroidal anti-inflammatory drugs and corticosteroids.

### Clinical interventions

All surgical procedures and clinical data collection were performed by a single experienced surgeon (I.A) who has 20 years of experience after graduating from his clinical residency in oral surgery and medicine. Prior to surgery, patients received all information about possible treatment options and signed a voluntary informed consent for surgery. Professional oral hygiene procedures were performed at 2 weeks before surgery. On the day of surgery, patients received a prophylactic dose of penicillin antibiotic (2 g amoxiclav, LEK, d.d., Slovenia) 1 h before the surgery. All patients were rinsed with 0.2% chlorhexidine solution (Corsodyl, GlaxoSmithKline) for 60s. Local anesthesia was performed using Ubistesin Forte (articaine hydrochloride 40 mg and epinephrine hydrochloride 0.006 mg, 3 M ESPE, St. Paul, MN, USA).

### Implant placement

The operation was performed as follows: in the area of the missing tooth, a linear incision was made along the most coronal part of the alveolar ridge, and in the area of the neighboring teeth, sulcular incisions were made. All implants placed in this study were of the same model (Astra Tech OsseoSpeed TX, Dentsply Implants Manufacturing GmbH, Germany) and were inserted after raising a full-thickness flap according to the standard surgical protocol. The internal surface of the implant was disinfected with a 0.2% chlorhexidine solution (Corsodyl, GlaxoSmithKline), followed by rinsing of the surgical site with sterile physiological saline. Subsequently, a healing abutment of standard size (diameter 4.5 mm, height 4 mm) was placed. Wound was sutured with two single sutures (Prolene 6.0, Johnson and Johnson, Norderstedt, Germany) around healing abutment.

### Radiographic assessment

At the 3-month healing stage, standardized periapical radiographs were obtained using the parallel technique and a digital radiovisiography system (EzSensor, VATECH). Marginal bone levels were measured mesially and distally from the implant shoulder to the first bone-to-implant contact, calibrated to the known implant length. The mean of mesial and distal measurements was used for analysis.

### Study group stratification

For the purpose of this study, implant mucosal tunnel depth was defined as the vertical distance between the peri-implant mucosal margin and the most coronal aspect of the implant platform prior to prosthetic restoration. This parameter reflects the depth of the peri-implant soft tissue tunnel formed following implant placement and healing and is independent of the contour of a definitive prosthetic restoration.

After a standardized healing period of three months, the depth of the implant mucosal tunnel was assessed following removal of the healing abutments. Measurements were obtained using a sterile UNC-15 graduated periodontal probe at four aspects of each implant site (buccal, oral, mesial, and distal). The vertical distance from the implant platform to the mucosal margin was recorded at each point, and the arithmetic mean of the four measurements, expressed in millimeters, was defined as the implant mucosal tunnel depth.

Based on these post-healing measurements, participants were stratified into three groups: less than 3 mm (Group 1), 3 to 5 mm (Group 2), and greater than 5 mm (Group 3). This grouping reflected natural anatomical variation of peri-implant soft tissue dimensions rather than investigator-driven assignment.

All participants were examined at a single post-healing time point. Microbiological sampling and TNF-α gene expression analysis were performed once for each implant site, and no longitudinal follow-up measurements were included in the present study.

**Fig. 1:**
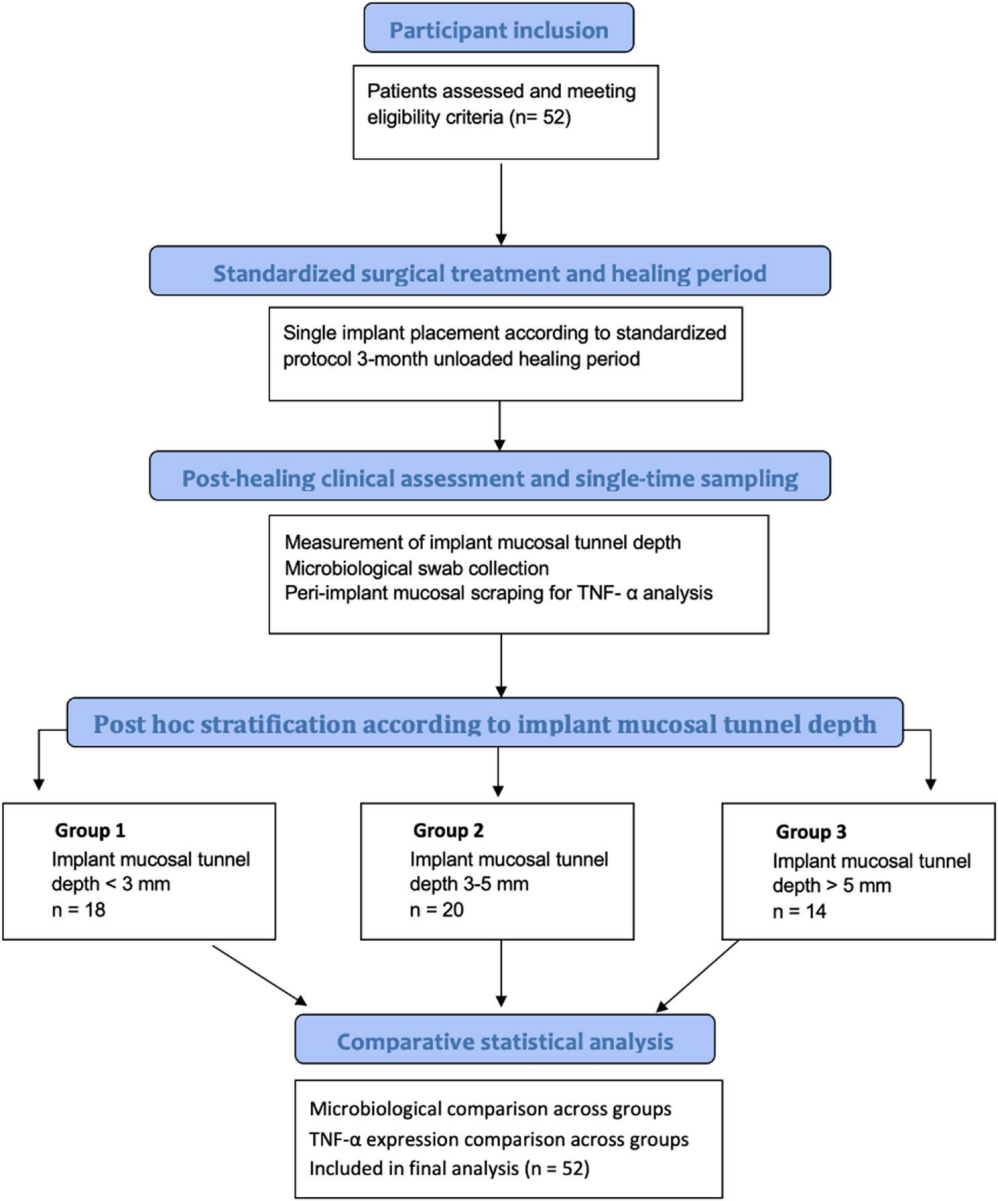
Participant inclusion and post-healing group stratification

The process of participant inclusion, post-healing assessment, and post hoc stratification according to implant mucosal tunnel depth is illustrated in Fig. 1.

### Evaluation of microbiota

**Fig. 2:**
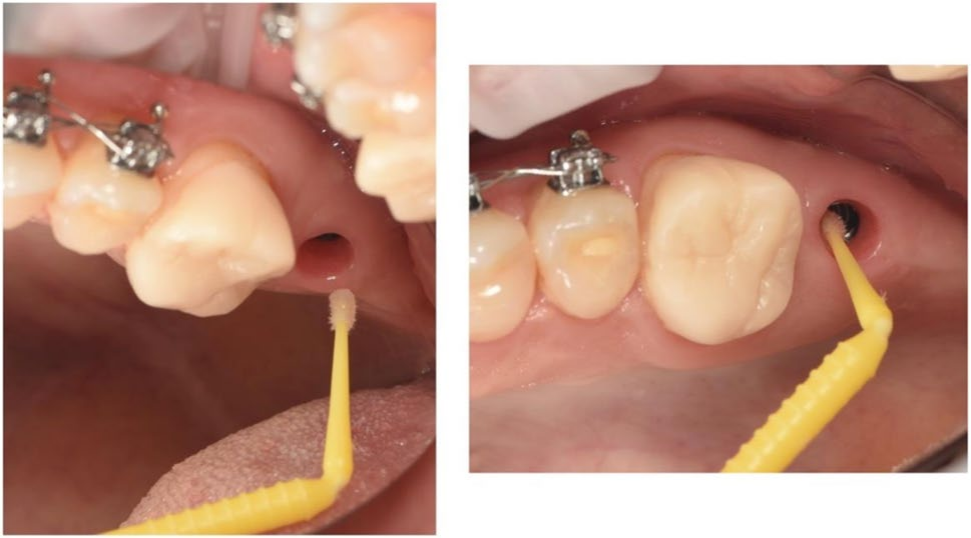
Swab collection from the inner surface of the implant mucosal tunnel

After a standardized unloaded healing period of 3 months, a single microbiological sampling procedure was performed at each implant site. Swabs were collected from the inner mucosal lining of the implant mucosal tunnel using sterile dental applicators (Euronda, Italy), while carefully avoiding contact with the implant internal connection surfaces in order to minimize contamination from non-mucosal areas (Fig. 2).

This sampling approach was intended to recover viable microorganisms colonizing the internal soft-tissue surface of the peri-implant mucosal tunnel.

Following swab collection, the applicator tip was aseptically cut with sterile scissors and placed into Amies transport medium (NUOVA APTACA S.R.L., Italy) for laboratory transport. Microbiological analysis was performed by blinded investigator (O. S.).

In the laboratory, microbiological samples were cultured on Petri dishes containing selective solid media. For the isolation of staphylococci, nutrient medium No. 10 GRM supplemented with egg yolk (State Research Center for Applied Microbiology, Obolensk, Russia) was used, whereas enterobacteria were cultured on Endo-GRM agar. Uropathogenic bacteria were isolated using UriSelect 4 medium, while fungi were grown on nutrient medium No. 2 GRM and Sabouraud agar (State Research Center for Applied Microbiology, Obolensk, Russia). In addition, nutrient GRM agar supplemented with 5% sterile defibrinated horse blood (ZAO Ecolab, Russia) was employed.

The plates were incubated for 24 to 48 h at 37 °C under both aerobic and anaerobic conditions, the latter established using an anaerobic jar with ANAEROGAS gas-generating packs (NIKI-MLT, Russia). Following incubation, colonies were counted, and aerobic as well as facultative anaerobic microorganisms were identified using standard physiological and biochemical tests. Pure cultures were obtained by subsequent subculturing.

Microbial identification was further performed by MALDI-TOF mass spectrometry (MALDI Biotyper Sirius RUO System, Bruker, Germany). Thus, the microbiological protocol was designed to characterize the cultivable viable fraction of the peri-implant microbiota rather than the complete microbial community. Briefly, a freshly isolated colony was transferred with a disposable loop onto a target plate well (MSP chip, Bruker) and air-dried. Microbial proteins were extracted by applying 1–2 µL of 70% formic acid (Sigma-Aldrich, USA), followed by addition of 1–2 µL of matrix solution consisting of α-cyano-4-hydroxycinnamic acid dissolved in 50% acetonitrile and 2.5% trifluoroacetic acid (Riedel-de Haën/Honeywell, Germany) for peptide ionization. The prepared plates were analyzed by mass spectrometry, and identification results were considered reliable when the database matching score was ≥ 2.0.

After incubation, bacterial growth was quantified by standard dilution procedures and expressed as colony-forming units. For descriptive comparison of the total microbial yield recovered from each implant site, results were recorded as CFU/swab. In addition, for semi-quantitative clinical interpretation, growth intensity was categorized according to conventional microbiological thresholds, with values ≥ 10⁵ CFU/mL considered indicative of clinically significant viable bacterial growth.

It should be noted that the applied culture-dependent approach primarily detects viable cultivable microorganisms and does not permit comprehensive characterization of non-cultivable, low-abundance, or fastidious taxa; therefore, microbiological findings should be interpreted as reflecting the cultivable component of the peri-implant microbial ecosystem.

All analyses were performed using equipment from the Shared Research Facility of the I.I. Mechnikov Research Institute of Vaccines and Sera (Moscow, Russia).

### Gene expression analysis of TNF-α

As a secondary exploratory outcome, the local expression level of the pro-inflammatory cytokine TNF-α was assessed in peri-implant mucosal scrapings obtained from the inner side of the implant mucosal tunnel using sterile dental brushes (Placontrol, INC, USA) (Fig. 2).

Total RNA was extracted from the samples using the commercial “RIBO-sorb” kit (AmpliSens, Russia) in accordance with the manufacturer’s instructions. Reverse transcription was subsequently performed with the OT-1 kit (Syntol, Russia) to obtain complementary DNA (cDNA). The resulting cDNA served as a template for quantitative analysis of *TNF-α* expression by real-time polymerase chain reaction (qPCR). Amplification reactions were carried out on a DT-96 thermal cycler (DNA-Technology, Russia) using primers and reagents supplied by Syntol (Russia).

The amplification protocol included an initial denaturation step at 95 °C for 3 min (1 cycle), followed by 40 cycles of denaturation at 95 °C for 20 s and combined annealing/extension at 62 °C for 40 s.

The obtained threshold cycle (Ct) values were normalized to the reference gene β-actin (ACTB) using the 2–ΔΔCt method, and the results were expressed in relative units (R.U.).

### Patient-related baseline characteristics

For all included patients, baseline demographic, behavioral, and periodontal-history related variables were recorded, including sex, residential region, toothbrushing habits, mouth rinsing, flossing, alcohol consumption, self-reported stress, previous periodontal treatment history, and gingival biotype.

### Statistical analyses

The statistical analysis was performed by an independent blinded investigator (A.A.). Data processing was carried out using Microsoft Office Excel 2019 and SPSS Statistics 27.0.1.0 (IBM, USA). Quantitative variables were described using median values (Me) and lower and upper quartiles (Q1–Q3) due to the non-normal distribution of the data.

Intergroup comparisons among the three observationally stratified study groups were performed using the non-parametric Kruskal–Wallis H test. To estimate the magnitude of intergroup differences, effect size for the Kruskal–Wallis test was additionally calculated using eta-squared (η²). When statistically significant overall differences were detected, pairwise comparisons were conducted using the Mann–Whitney U test with Bonferroni-adjusted significance levels (*p* < 0.017).

These analyses were applied, as appropriate, to microbiological growth variables, cultivable species diversity indices, and TNF-α gene expression values.

Given the exploratory cross-sectional design of the study and the absence of prior effect estimates for this specific biological model, the statistical findings should be interpreted as hypothesis-generating rather than confirmatory.

## Results

### Participant characteristics and observational stratification

Patient recruitment was conducted from October 2021 to December 2022. Fifty-two patients (mean age 51.5 ± 6.6 years) who met the eligibility criteria and completed the standardized 3-month healing phase were included in the observational analysis.

Based on the measured implant mucosal tunnel depth after healing, patients were observationally stratified into Group 1 (18 patients; mean age 50.1 ± 6.4 years), Group 2 (20 patients; mean age 52.6 ± 7.0 years), and Group 3 (14 patients; mean age 51.9 ± 6.3 years).

The groups were comparable in terms of age and gender distribution (*p* = 0.705 and *p* = 0.054, respectively) (Table 1).

**Table 1:** Baseline characteristics

	All patients *n* = 52	Group 1 (< 3 mm) *n* = 18	Group 2 (3–5 mm) *n* = 20	Group 3 (> 5 mm) *n* = 14	*p*-Value
Age (years)	51,5 ± 6,653 (46–55)	50,1 ± 6,453 (43–55)	52,6 ± 7,055(46–58,5)	51,9 ± 6,352 (48–56)	0,705
Gender					0,054
Female	29 (55,8%)	10 (55,6%)	12 (60%)	7 (50%)	
Male	23 (44,2%)	8 (44,4%)	8 (40%)	7 (50%)	
Implant position					0,083
First molar	41(78,8%)	14 (77,8%)	14 (70,0%)	13 (92,9%)	
Second molar	11(21,2%)	4 (22,2%)	6 (30,0%)	1 (7,1%)	
Implant length (mm)					0,178
8	14 (25,0%)	5 (27,8%)	6 (30,0%)	3 (21,4%)	
9	38 (75,0%)	13 (72,2%)	14 (70,0%)	11 (78,6%)	
Implant diameter (mm)	4,0 (100,0%)	4,0 (100,0%)	4,0 (100,0%)	4,0 (100,0%)	1,0
Bone resorption (mm)		0,71 ± 0,81 0,43 (0–1,3)	0,28 ± 0,5 0 (0–0,43)	0,17 ± 0,48 0 (0–0)	0,072
Keratinized mucosa width (mm)		3,47 ± 0,72 3 (3–4)	3,54 ± 1,01 3 (3–4)	3,69 ± 0,84 4 (3–4)	0,262

The mean implant mucosal tunnel depth in Groups 1, 2, and 3 was 1.98 ± 0.49 mm, 3.55 ± 0.48 mm, and 5.55 ± 1.15 mm, respectively (p < 0.001), confirming clear separation of the three observational subgroups

### Results of microbiological analysis

A total of 52 microbiological samples were obtained from 52 patients, yielding 262 cultivable microbial isolates representing 46 species from 15 genera.

**Fig. 3:**
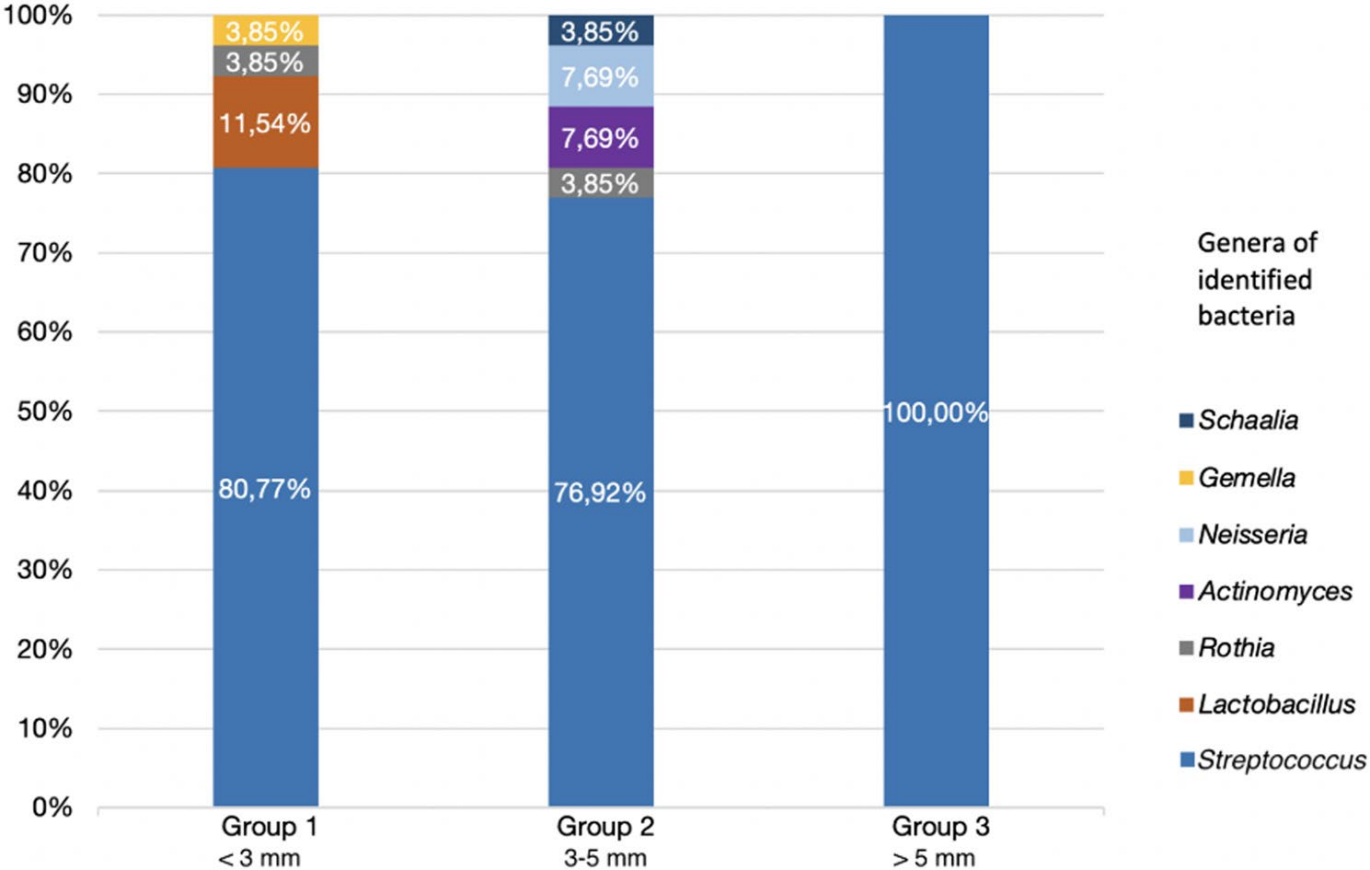
Distribution of cultivable microorganisms with clinically significant growth (≥ 10⁵ CFU/mL) across study groups. Intergroup differences were assessed using the Kruskal–Wallis test

Species diversity among cultivable microorganisms demonstrating clinically significant growth (≥ 10⁵ CFU/mL) varied according to implant mucosal tunnel depth (Fig. 3).

The highest Shannon diversity index (H), calculated for cultivable microorganisms with clinically significant growth, was observed in Group 2 (H = 0.847), whereas Group 1 demonstrated lower diversity (H = 0.672; *p* = 0.012). In contrast, Group 3 (> 5 mm) demonstrated markedly reduced cultivable diversity, with clinically significant growth represented predominantly by streptococci, resulting in a Shannon index of 0.00.

Among all isolated cultivable microorganisms, facultative anaerobic species predominated (81.68%), while aerobic microorganisms accounted for 18.32%. This general distribution pattern remained comparable among all study groups (Table 2).

**Table 2:** Distribution of aerobic and facultative anaerobic cultivable microorganisms, %

		Groups		
	Total count	Group 1 (< 3 mm)	Group 2 (3–5 mm)	Group 3 (> 5 mm)
Aerobes	18,32%	16,47%	18,49%	20,69%
Facultative Anaerobes	81,68%	83,53%	81,51%	79,31%

**Fig. 4:**
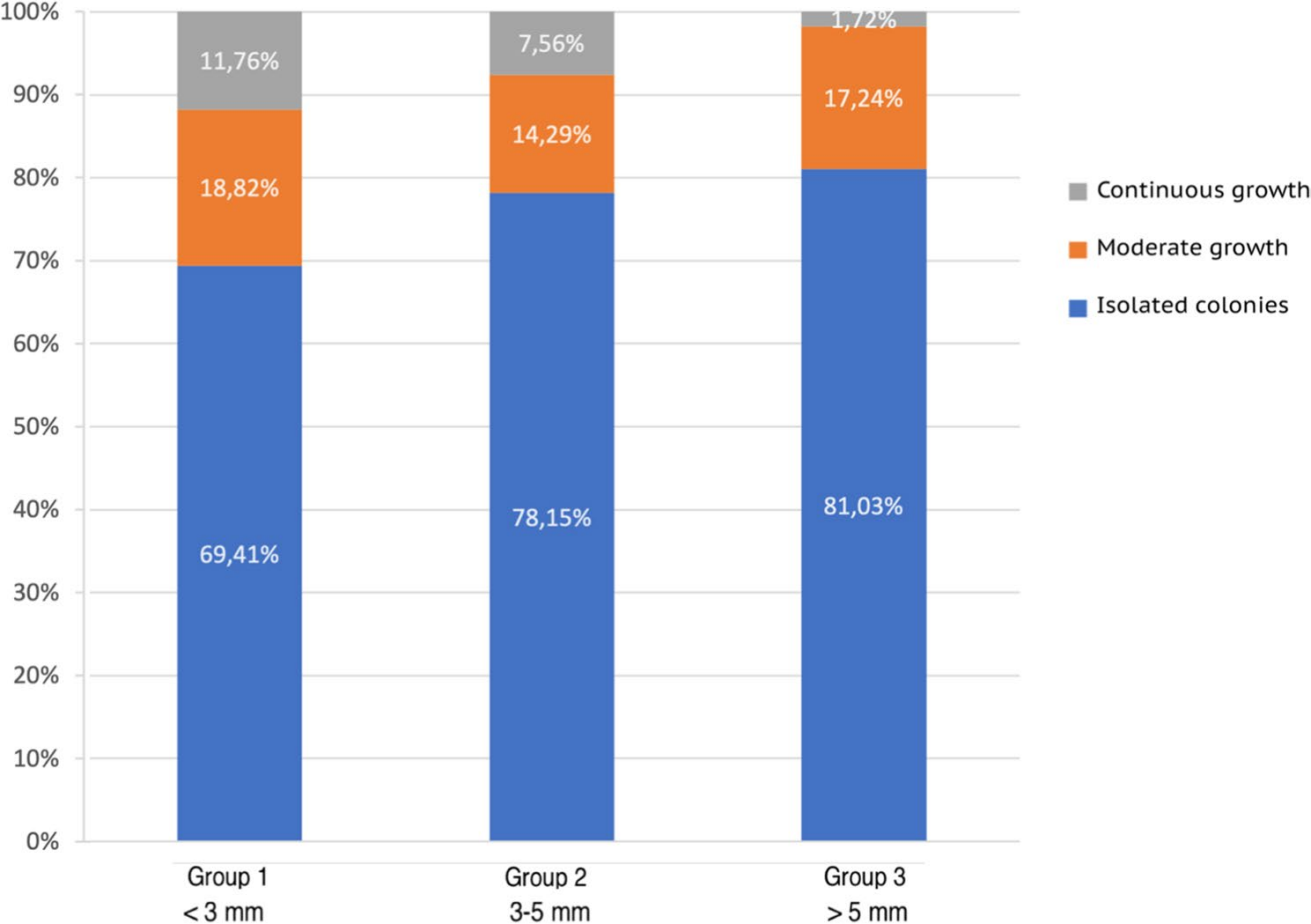
Distribution of cultivable microbial growth intensity according to implant mucosal tunnel depth. Group 1 (< 3 mm); Group 2 (3–5 mm); Group 3 (> 5 mm). *p < 0.017, Mann–Whitney U test

Cultivable microbial growth intensity also demonstrated significant dependence on implant mucosal tunnel depth (*p* = 0.0284). Specifically, the proportion of samples exhibiting confluent or abundant bacterial growth (≥ 10⁷ CFU/mL) progressively decreased as tunnel depth increased. In Group 1, 11.76% of samples demonstrated confluent growth, whereas in Group 3 this growth pattern was observed only in 1.72% of samples (Fig. 4).

Table 3 presents the prevalence of the predominant cultivable microorganisms detected across the three groups.

**Table 3:** Frequency distribution of predominant cultivable microorganisms across study groups (% of total group isolates, CFU/mL)

Microorganisms	Microbial load, CFU/mL Me (Q1-Q3)		
	Group 1 (< 3 mm)	Group 2 (3–5 mm)	Group 3 (> 5 mm)
*Actinomyces*	10 (4–10) × 10^5^	10 (2–50) × 10^5^	6 (5–7,75) × 10^5^
*Gemella*	8 (5-245) × 10^5^	10 (3,5-12.5) × 10^5^	5 (5–10) × 10^5^
*Neisseria*	3 (2–20) × 10^5^	3 (1–20) × 10^5^	8 (4,5–10) × 10^5^
*Rothia*	10 (5,5–26,5) × 10^5^	4 (2–15) × 10^5^	8 (4–10) × 10^5^
*Schaalia*	5,5 (3,25 − 7,75) × 10^5^	3.5 (1–6,25) × 10^5^	5 (4–17,5) × 10^5^
*Streptococcus*	100 (20–275) × 10^5^	25 (10–100) × 10^5^	25 (7-162,5) × 10^5^

Streptococcus spp. were cultured from all samples irrespective of implant mucosal tunnel depth. Gemella spp. demonstrated a higher prevalence in Group 3 (40.0%) than in Group 1 (11.1%) (*p* = 0.0410). Schaalia odontolytica was more frequently detected in Group 2 (40.0%) than in Group 1 (11.1%) (*p* = 0.0673). In addition, the prevalence of Rothia spp. gradually decreased with increasing tunnel depth, from 77.7% in Group 1 to 60.0% in Group 2 and 40.0% in Group 3

A more detailed analysis of Streptococcus species composition showed that deeper implant mucosal tunnels tended to demonstrate higher frequencies of Str. anginosus (28.57%), Str. oralis (21.43%), and Str. gordonii (14.29%), although these differences did not reach statistical significance. Str. infantis was detected only in Group 1, whereas Str. parasanguinis was not identified in Group 3 and Str. pneumoniae was absent in groups with tunnel depth greater than 3 mm.

Overall, increasing implant mucosal tunnel depth was associated with reduced cultivable species diversity and lower intensity of cultivable bacterial growth under the applied laboratory conditions. These findings indicate that reduced cultivable diversity was accompanied by a decrease in high-density bacterial growth, suggesting a simplified rather than expanded microbial profile.

### Results of TNF-α gene expression analysis

**Fig. 5:**
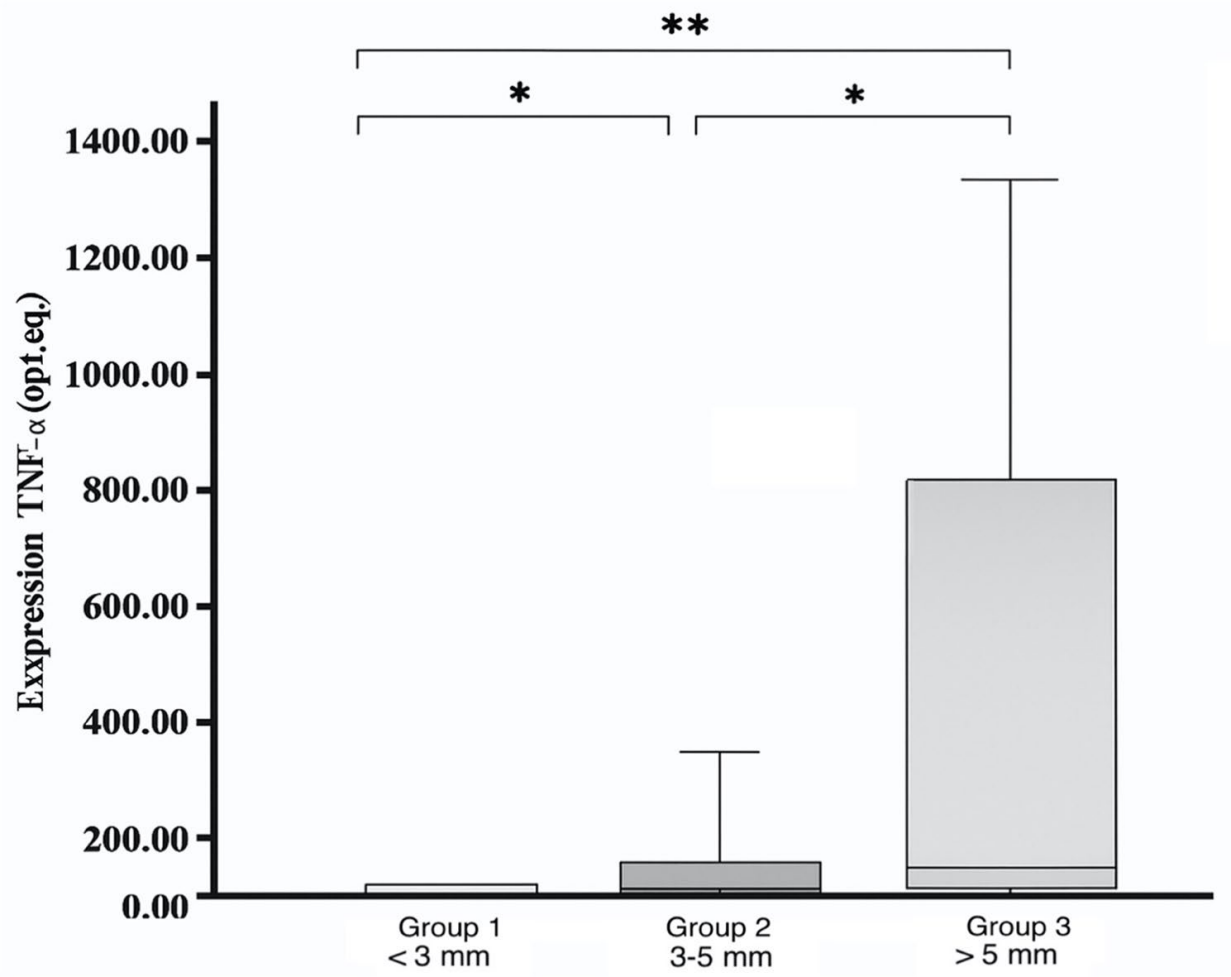
Median TNF-α gene expression levels (relative units) according to implant mucosal tunnel depth. Overall group differences were assessed using the Kruskal–Wallis test; pairwise comparisons were performed using the Mann–Whitney U test with Bonferroni correction (*p < 0.05; **p < 0.017)

A progressive increase in TNF-α gene expression was observed with increasing implant mucosal tunnel depth (Fig. 5).

Median TNF-α expression values were 1.15 (0.53–2.42) in Group 1, 7.73 (3.50–39.40) in Group 2, and 43.86 (13.23–610.66) in Group 3.

Compared with Group 1, TNF-α expression demonstrated an approximately 7-fold increase in Group 2 (*p* < 0.017) and an approximately 38-fold increase in Group 3 (*p* < 0.017).

The estimated effect size for TNF-α expression across groups, calculated using eta-squared (η²), was approximately 0.13, indicating a moderate-to-large magnitude of intergroup difference despite the exploratory sample size.

Thus, greater implant mucosal tunnel depth was consistently associated with increased local TNF-α expression. However, due to the absence of concurrent peri-implant clinical inflammatory measurements at the time of sampling, these findings should be interpreted as biologic associations rather than direct evidence of active peri-implant inflammatory disease.

## Discussion

In the present observational study, differences in the cultivable peri-implant microbiota were identified depending on implant mucosal tunnel depth. Therefore, the first null hypothesis was rejected. At the same time, a predominance of cultivable periodontal pathogenic microorganisms was not observed in implants with a deep mucosal tunnel (> 5 mm); thus, the second null hypothesis was not supported under the microbiological conditions applied in this investigation.

According to several authors, accessibility for effective biofilm removal around implant-supported restorations is crucial for the prevention and treatment of peri-implant inflammatory diseases [14, 16]. Numerous clinical and experimental studies have demonstrated a direct relationship between plaque accumulation around implants and the onset of peri-implant mucositis or peri-implantitis [32]. Since microorganisms are considered a principal etiologic factor in these conditions, it has been hypothesized that increased implant mucosal tunnel depth may create a protected ecological niche favoring microbial accumulation and altered peri-implant biofilm composition.

In the present study, the diversity of cultivable microorganisms demonstrating clinically significant growth differed among patients with varying implant mucosal tunnel depths. Specifically, the highest Shannon diversity index was observed in Group 2, whereas Group 3 (> 5 mm) demonstrated clinically significant growth represented predominantly by streptococci. In addition, increasing implant mucosal tunnel depth was associated with a reduction in the proportion of samples showing confluent bacterial growth. These findings suggest that deeper peri-implant soft tissue tunnels may be associated not with increased abundance of cultivable pathogens, but rather with a simplified cultivable microbial profile.

A substantial proportion of Streptococcus representatives belongs to the yellow cluster according to the classification of Socransky S., which is generally considered to have a limited role in periodontal tissue destruction. Previous studies also indicate that streptococci are frequently detected in clinically healthy peri-implant and periodontal sites and are not regarded as specific biomarkers of peri-implant inflammation [33, 34]. Although isolated colonies of other genera were also cultured, their prevalence remained comparatively low.

Notably, Gemella spp. were identified more frequently in Group 3 than in Group 1. Although isolated reports have described an increased prevalence of Gemella in peri-implantitis lesions [35], this genus is not consistently regarded as a major contributor to destructive peri-implant inflammatory disease. Therefore, within the limits of cultivable microbiological analysis, the present results do not support the assumption that increasing implant mucosal tunnel depth is necessarily associated with dominance of cultivable periodontal pathogens.

An important contextual consideration is that all enrolled participants demonstrated satisfactory oral hygiene and were included only after strict clinical screening. Previous literature indicates that patients with adequate plaque control may maintain peri-implant tissue stability even in the presence of deeper peri-implant sulci, whereas compromised hygiene may substantially increase the retention of biofilm in anatomically inaccessible areas [14]. Consequently, deep peri-implant soft tissue architecture may be clinically relevant primarily in conjunction with additional modifying factors rather than as an isolated determinant.

For this reason, the present findings should not be interpreted as evidence that deep implant mucosal tunnel depth is either protective or predictive of future peri-implant disease. Rather, under controlled hygiene conditions and within the short-term healing period assessed, increased mucosal tunnel depth was associated with differences in cultivable microbial composition without demonstrable predominance of dominant cultivable periodontal pathogens.

To complement conventional clinical and radiographic assessment of peri-implant tissues, several studies have investigated non-invasive biologic markers, including cytokine profiling, inflammatory mediator analysis, and molecular diagnostic approaches [31–34]. In addition to microbiological assessment, the present study included TNF-α gene expression analysis as a secondary biologic endpoint in order to explore whether peri-implant soft tissue tunnel depth may also be associated with changes in local host immune activity. TNF-α is a multifunctional pro-inflammatory cytokine involved in inflammatory regulation, bone remodeling, and innate immune responses to bacterial stimulation [28, 29].

A sequential increase in TNF-α expression was observed with increasing implant mucosal tunnel depth (Fig. 5). Median expression in Group 2 was approximately seven-fold higher than in Group 1, whereas Group 3 demonstrated a median increase of approximately thirty-eight-fold relative to Group 1. The calculated η² effect size indicated a moderate-to-large magnitude of intergroup difference. These findings suggest that peri-implant soft tissue architecture may influence not only the cultivable microbial profile but also the biologic activity of peri-implant tissues.

However, this observation requires cautious interpretation. Site-specific peri-implant clinical inflammatory parameters, including probing depth, bleeding on probing, and mucosal inflammation scores, were not recorded at the exact time of microbiological and cytokine sampling. Therefore, direct clinical correlation between elevated TNF-α expression and contemporaneous inflammatory status cannot be established within this study. The cytokine findings should thus be interpreted as biologic observations rather than as clinically validated indicators of active peri-implant inflammation.

The apparent discrepancy between limited recovery of dominant cultivable pathogens and elevated TNF-α expression may reflect the multifactorial nature of peri-implant host responses. First, culture-based microbiological methods identify only viable and cultivable organisms and may underestimate low-abundance or non-cultivable taxa capable of stimulating immune signaling. Several sequencing-based studies have shown substantially greater taxonomic complexity in peri-implant biofilms than is detectable by conventional culturing methods. Second, TNF-α upregulation may also be influenced by non-microbial stimuli, including host–implant interactions, microgaps at the implant–abutment interface, titanium particle exposure, or mechanical irritation within deeper peri-implant soft tissue tunnels. Therefore, elevated cytokine activity in the absence of dominant cultivable pathogens should not be considered biologically implausible.

The present study has several limitations that must be emphasized. First, this was an observational cross-sectional investigation with post hoc stratification according to naturally formed implant mucosal tunnel depth rather than a randomized clinical trial; therefore, causal inferences cannot be made. Second, the total cohort of 52 patients, subdivided into three unequal groups, limits statistical power and generalizability, and no formal a priori sample size calculation was performed because of the exploratory design and lack of prior effect estimates for this biological model. Third, implant mucosal tunnel depth was measured clinically by a single examiner after healing, and no formal calibration or intra-examiner reproducibility analysis was performed, which may introduce measurement variability and potential bias in the primary independent variable. Fourth, microbiological analysis was limited to culture-based methods with MALDI-TOF identification of dominant viable isolates and therefore does not represent the full peri-implant microbiome. Fifth, peri-implant clinical inflammatory indices were not recorded concurrently with biologic sampling, limiting clinical interpretation of TNF-α changes. Finally, the study evaluated only an early three-month healing interval and does not provide longitudinal evidence regarding future peri-implant disease development.

Future investigations should therefore include longitudinal observational or randomized clinical designs with larger and more balanced cohorts, calibrated measurements of peri-implant soft tissue dimensions, simultaneous recording of clinical inflammatory indices, and sequencing-based microbiome characterization together with expanded cytokine profiling in order to better define the biologic significance of peri-implant soft tissue tunnel depth.

## Conclusion

Within the limitations of this exploratory observational study, implant mucosal tunnel depth appeared to be associated with measurable differences in cultivable microbial composition and local TNF-α expression in clinically stable peri-implant tissues.

Implants with deeper mucosal tunnels (> 5 mm) demonstrated reduced cultivable species diversity and lower cultivable bacterial growth intensity, without a consistent predominance of cultivable classical periodontal pathogens under the applied laboratory conditions.

At the same time, increased TNF-α expression was observed with increasing tunnel depth, suggesting altered local biologic host activity. However, in the absence of contemporaneous peri-implant clinical inflammatory measurements and longitudinal follow-up, these cytokine findings should not be interpreted as evidence of active disease or future peri-implant pathology.

Accordingly, the present results should be regarded as exploratory biologic associations that require confirmation in larger longitudinal studies using comprehensive clinical and sequencing-based microbiologic assessment.

## Data Availability

The datasets used and/or analyzed during the current study are available from the corresponding author on reasonable request.

## Abbreviations

MALDI-TOF: Matrix-Assisted Laser Desorption/Ionization Time-of-Flight
TNF-α: Tumor Necrosis Factor alpha
PCR: Polymerase Chain Reaction
ASA: American Society of Anesthesiologists
FMBS: Full-Mouth Bleeding Score
FMPS: Full-Mouth Plaque Score
NSAIDs: Non-Steroidal Anti-Inflammatory Drugs
RNA: Ribonucleic Acid
cDNA: Complementary Deoxyribonucleic Acid
qPCR: Quantitative Polymerase Chain Reaction
Ct: Threshold Cycle
ACTB: Beta-actin (reference gene)
CFU/mL: Colony-Forming Units per Milliliter
H: Shannon Diversity Index
